# Hypomethylation and overexpression of ITGAL (CD11a) in CD4^+^ T cells in systemic sclerosis

**DOI:** 10.1186/1868-7083-6-25

**Published:** 2014-11-11

**Authors:** YaoYao Wang, Ye Shu, YangFan Xiao, Qing Wang, Takuro Kanekura, YaPing Li, JiuCun Wang, Ming Zhao, QianJin Lu, Rong Xiao

**Affiliations:** Department of Dermatology, Second Xiangya Hospital, Central South University, 139 Ren-Min Road, Changsha, 410011 China; Department of Dermatology, Sir Run Run Shaw Hospital, Zhejiang University, 3 East Qingchun Road, Hangzhou, 310016 China; Department of Dermatology, Hunan Children’s Hospital, 86 Zi-Yuan Road, Changsha, 410007 China; Department of Dermatology, Kagoshima University Graduate School of Medical and Dental Sciences, 8-35-1 Sakuragaoka, Kagoshima, 890-8520 Japan; Ministry of Education (MOE) Key Laboratory of Contemporary Anthropology and State Key Laboratory of Genetic Engineering, School of Life Sciences, Fudan University, 220 Handan Road, 200433 Shanghai, China; Hunan Key Laboratory of Medical Epigenomics, 139 Ren-Min Road, Changsha, 410011 China

**Keywords:** CD11a, CD4^+^ T cells, COL1A2, DNA methylation, Systemic sclerosis

## Abstract

**Background:**

The pathogenesis and etiology of systemic sclerosis (SSc) are complex and poorly understood. To date, several studies have demonstrated that the activation of the immune system undoubtedly plays a pivotal role in SSc pathogenesis. Activated immune effector T cells contribute to the release of various pro-inflammatory cytokines and drive the SSc-specific autoantibody responses. This, and a profibrotic environment, are all-important components of abnormal active immune responses that can lead to pathological disorders of SSc. CD11a is essential to inflammatory and immune responses, regulating adhesive and co-stimulatory interactions between CD4^+^ T cells and other cells. Although CD11a is overexpressed in SSc patients, the mechanisms leading to this overexpression and its consequences remain unclear. DNA methylation, a main epigenetic modification, plays an important role in the regulation of gene expression and is involved in the pathogenesis of autoimmune diseases. This work aims to investigate the effect of DNA demethylation on CD11a expression in SSc CD4^+^ T cells and to determine its functional significance. CD11a expression was measured using RT-PCR and flow cytometry. Bisulfite sequencing was used to determine the methylation status of the CD11a regulatory region. CD4^+^ T cells were co-cultured with antigen-presenting cells, B cells, or fibroblasts with and without anti-CD11a, and proliferation of CD4^+^ T cells, IgG production by B cells, and expression levels of COL1A2 mRNA by fibroblasts were evaluated.

**Results:**

Elevated CD11a expression levels were observed in CD4^+^ T cells from SSc patients; these levels were found to be positively correlated with disease activity. The methylation levels of the CD11a regulatory sequences were lower in SSc patients than in controls and inversely correlated with CD11a mRNA expression. Treatment of CD4^+^ T cells with 5-azacytidine (5-azaC) decreased CD11a promoter methylation and caused CD11a overexpression. SSc CD4^+^ T cells and 5-azaC-treated CD4^+^ T cells showed increased proliferation of CD4^+^ T cells, increased production of IgG by co-cultured B cells, and induced expression of COL1A2 mRNA by co-cultured fibroblasts. These stimulatory effects were abrogated by anti-CD11a.

**Conclusions:**

Demethylation of CD11a regulatory elements and subsequent CD11a overexpression in CD4^+^ T cells may mediate immunological abnormalities and fibrotic processes in SSc.

## Background

Systemic sclerosis (SSc) is a complicated multisystem autoimmune connective tissue disease characterized by severe and extensive fibrosis of the skin and other internal organs, pronounced pathological changes in the microvasculature, and numerous immunological abnormalities [1, 2]. The etiopathogenesis of the disease is currently unknown. Historically, most research has focused on fibroblasts as pathogenic mediators of SSc [3]. However, in recent years, it has become readily apparent that the immune system, especially T lymphocytes, are more central to the process than previously thought [4]. Interaction of abnormally activated T cells with B cells, vascular endothelial cells, and fibroblasts partially drives the autoantibody responses, vascular endothelial injury, and tissue fibrosis that can explain the main features of SSc [5, 6].

CD11a, which is encoded by ITGAL, is an α-chain subunit of the lymphocyte function-associated antigen-1 (LFA-1, CD11a/CD18) molecule. Interaction through cell surface LFA-1 and ICAM-1, -2, or -3 provides an essential signal for the linkage between T cells and different cell types; this is important in inflammatory and immune responses [7, 8]. Elevated CD11a expression at the surface of the activated CD4^+^ T cell has been observed in peripheral blood, skin lesions, and gastric mucosa of patients with SSc [9, 10]. High levels of soluble ICAM-1 have been detected in the serum and overexpression of ICAM-1 in some activated immune cells, dermal endothelial cells, and fibroblasts of patients with SSc [10–13]. Blockage of the interactions between LFA-1 and ICAM-1 was found to significantly reduce the synthesis and secretion of autoantibodies and relieves cutaneous fibrosis in an experimental bleomycin-induced SSc model [14]. In this way, CD11a may participate in the pathogenesis of SSc. However, the mechanism underlying the overexpression of CD11a and its functional consequences in SSc is largely unknown and remains to be elucidated.

While some predisposing genetic factors have been identified in SSc [15], similar concordance rates have been observed in monozygotic (4.2%) and dizygotic twins (5.6%) in a cross-sectional study [16]. Genetic factors associated with SSc susceptibility have been unable to explain the full etiology of SSc. Incomplete concordance between identical twins implies that other, non-genetic factors are also involved in the pathogenesis of SSc. Recent studies have demonstrated that the epigenetic disruption of gene expression plays an equally important role in the development of SSc through interaction with our environment [17–20]. Evidence that has accumulated in recent years has shown that DNA methylation plays a fundamental role in gene regulation by modifying chromatin structure, affecting accessibility of transcription factors to their target binding motifs. In general, DNA methylation in the regulatory sequence results in changes in the chromatin configuration from an open, transcriptionally active status to a more compact, inactive configuration that is inaccessible to transcription factors and to the transcription machinery. Conversely, demethylation of promoters restores the transcriptionally permissive chromatin configuration and thus increases accessibility of RNA polymerase II and related transcription factors to DNA transcription sites, leading to transcriptional activation. DNA methylation inhibitors have previously been shown to cause demethylation of several methylation-sensitive genes, including CD11a. It also doubles their level of expression in normal CD4^+^ T cells [21]. CD11a overexpression in CD4^+^ T cells can induce major-histocompatibility-complex-specific T cell autoreactivity *in vitro* and autoimmunity *in vivo*, suggesting that the correlation of DNA hypomethylation and autoimmune diseases is due to the elevated level of CD11a [22, 23].

The CD11a expression levels and methylation status of the CD11a promoter region in CD4^+^ T cells were compared in SSc patients and healthy controls. More CD11a expression was observed in CD4^+^ T cells from patients with SSc than in controls, and the level of expression was found to be positively correlated with disease activity. The methylation levels of the DNA regulatory sequences of CD11a were lower in SSc patients than in controls, contributing to the high level of CD11a expression. The changes in methylation of CD11a were found to influence CD4^+^ T cell function and to be responsible for the pathogenesis of SSc. Current findings indicate that CD11a promoter hypomethylation can drive the onset and progress of SSc. These results may provide insight into the epigenetics, diagnosis, and treatment of SSc.

## Results

### CD11a expression is higher in CD4^+^ T cells from SSc patients than in those from controls

**Figure 1:**
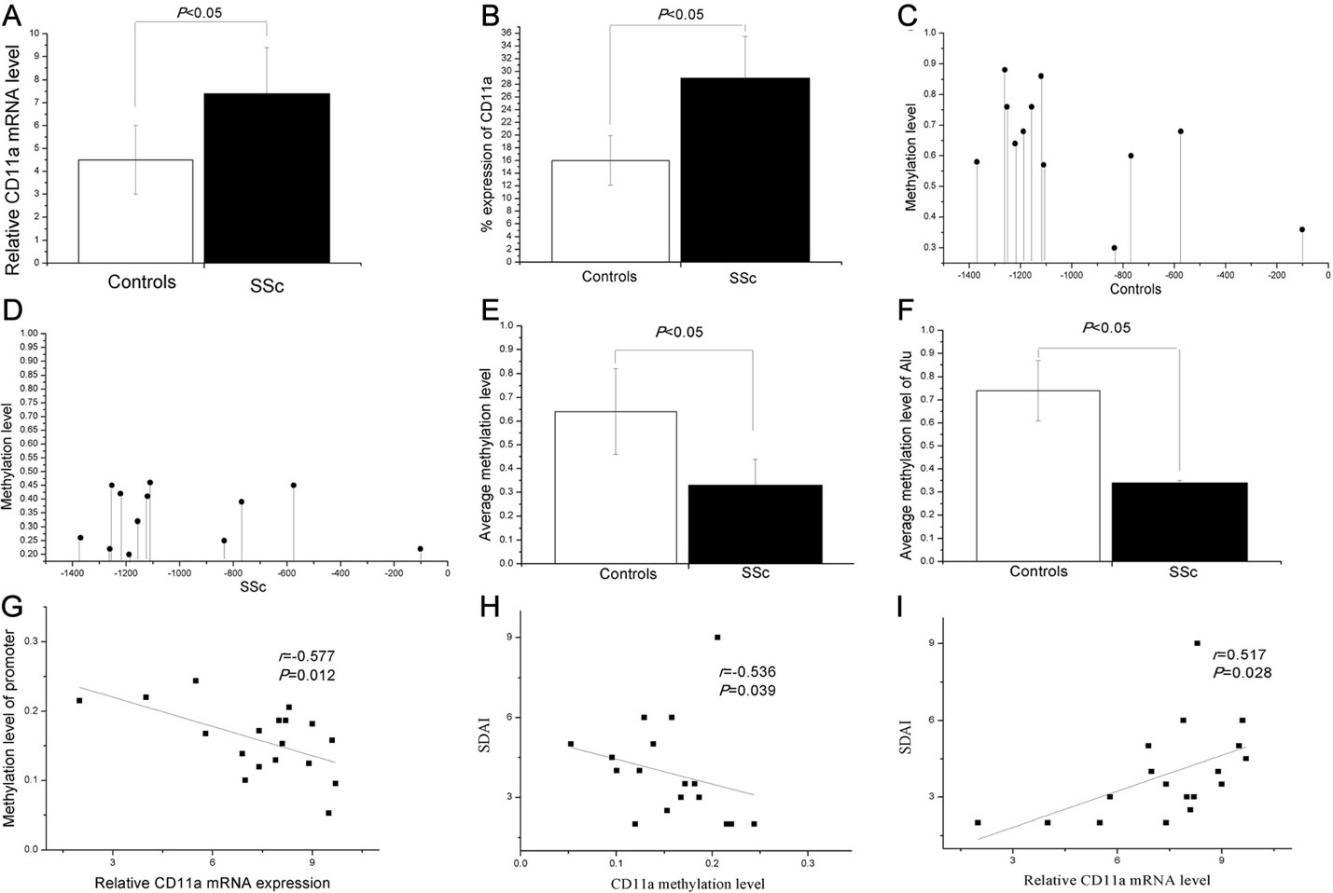
**Expression of CD11a and methylation status of the CD11a promoter region in CD4** ^**+**^ **T cells from patients with SSc.** (**A** and **B**) Elevated CD11a **(A)** mRNA and **(B)** protein expression in CD4^+^ T cells from patients with SSc. The methylation pattern of CD11a promoter region in CD4^+^ T cells from 15 healthy controls **(C)** and 18 SSc patients **(D)**. **(E)** The average methylation status of the 23 CG pairs was found to be significantly lower in SSc CD4^+^ T cells than in healthy controls. **(F)** The average methylation levels of the Alu element were also significantly lower in SSc CD4^+^ T cells than in healthy controls. (**G** and **H**) The average DNA methylation levels of the CD11a promoter were inversely correlated with the **(G)** relative mRNA level and **(H)** disease activity (Valentini scleroderma disease activity index; SDAI) in patients with SSc. **(I)** There was a significant positive correlation between individual relative CD11a mRNA expression levels (X-axis) and SDAI (Y-axis) in SSc patients.

Real-time quantitative RT-PCR analysis showed CD11a mRNA expression to be significantly more pronounced in SSc patients than in controls (7.4 ± 2.0 vs. 4.5 ± 1.3, *P* <0.05, Figure 1A). Two-color flow cytometry experiments showed the relative number of CD4^+^ CD11a^+^ T cells to be significantly higher in SSc patients than in healthy controls (29 ± 6.5% vs. 16 ± 3.9%, *P* <0.05, Figure 1B).

### Regulatory elements of CD11a promoter were hypomethylated in CD4^+^ T cells from SSc patients

**Figure 2:**
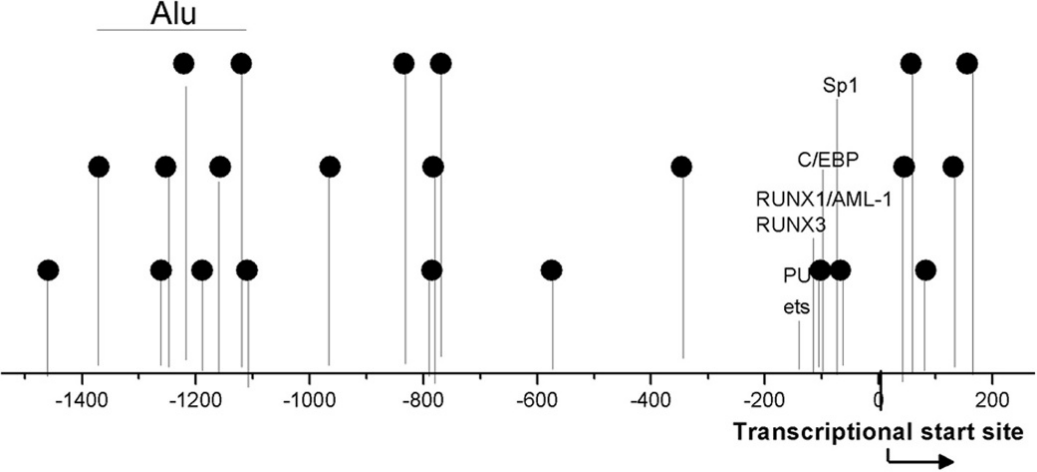
**Schematic representation of the CD11a gene promoter locus.** The 23 potentially methylated CpG pairs in the CD11a promoter sequences are identified by the lollipop-shaped figures. The region including Alu elements is denoted by the horizontal line. The transcriptionally relevant PU1, C/EBP, Sp1 transcription factor, ets-related factor, PEBP2/CBF/AML family of transcription factor, RUNX3, and RUNX1/AML-1 transcription factor binding sites and the transcription start site are also shown.

To investigate the level of DNA methylation of CD11a regulatory elements in SSc CD4^+^ T cells, the methylation status of 23 CG pairs in 1,699 bp of the CD11a gene promoter (-1486 to +213) containing the Alu elements, transcription factor binding sites and transcription start site was analyzed using bisulfite genomic DNA sequencing (Figure 2). The promoter fragments of the CD11a locus were amplified by PCR and amplified fragments were then cloned; 10 clones from each amplified fragment from each subject were sequenced. The methylation patterns of this region in CD4^+^ T cells from 15 healthy controls and 18 SSc patients are shown in Figure 1C,D. The average methylation status of the 23 CG pairs was observed to be significantly lower in SSc CD4^+^ T cells than in healthy controls (*P* <0.05, Figure 1E). The average methylation levels of the Alu element were also significantly lower in SSc CD4^+^ T cells than in healthy controls (*P* <0.05, Figure 1F).

### Correlation between CD11a promoter methylation levels and CD11a mRNA expression in SSc CD4^+^ T cells

We then analyzed the relationship between methylation levels in the CD11a promoter region and CD11a mRNA expression in CD4^+^ T cells of SSc patients. The mRNA levels of CD11a were negatively correlated with the mean methylation status of the 23 CG pairs in the promoter region in SSc patients (*r* = -0.577, *P* =0.012, Figure 1G).

### Correlations between disease activity and CD11a promoter methylation or CD11a expression level in SSc

The relationship between SSc disease activity, CD11a expression, and methylation of its promoter was evaluated. As shown in Figure 1H,I, Valentini scleroderma disease activity indexes (SDAIs) of SSc patients were inversely correlated with CD11a promoter methylation and positively correlated with the level of expression of CD11a mRNA (*r* = -0.536, *P* =0.039 and *r* =0.517, *P* =0.0285, respectively).

### Treatment with 5-azaC decreased CD11a regulatory sequence methylation and increased CD11a expression in normal CD4^+^ T cells

**Figure 3:**
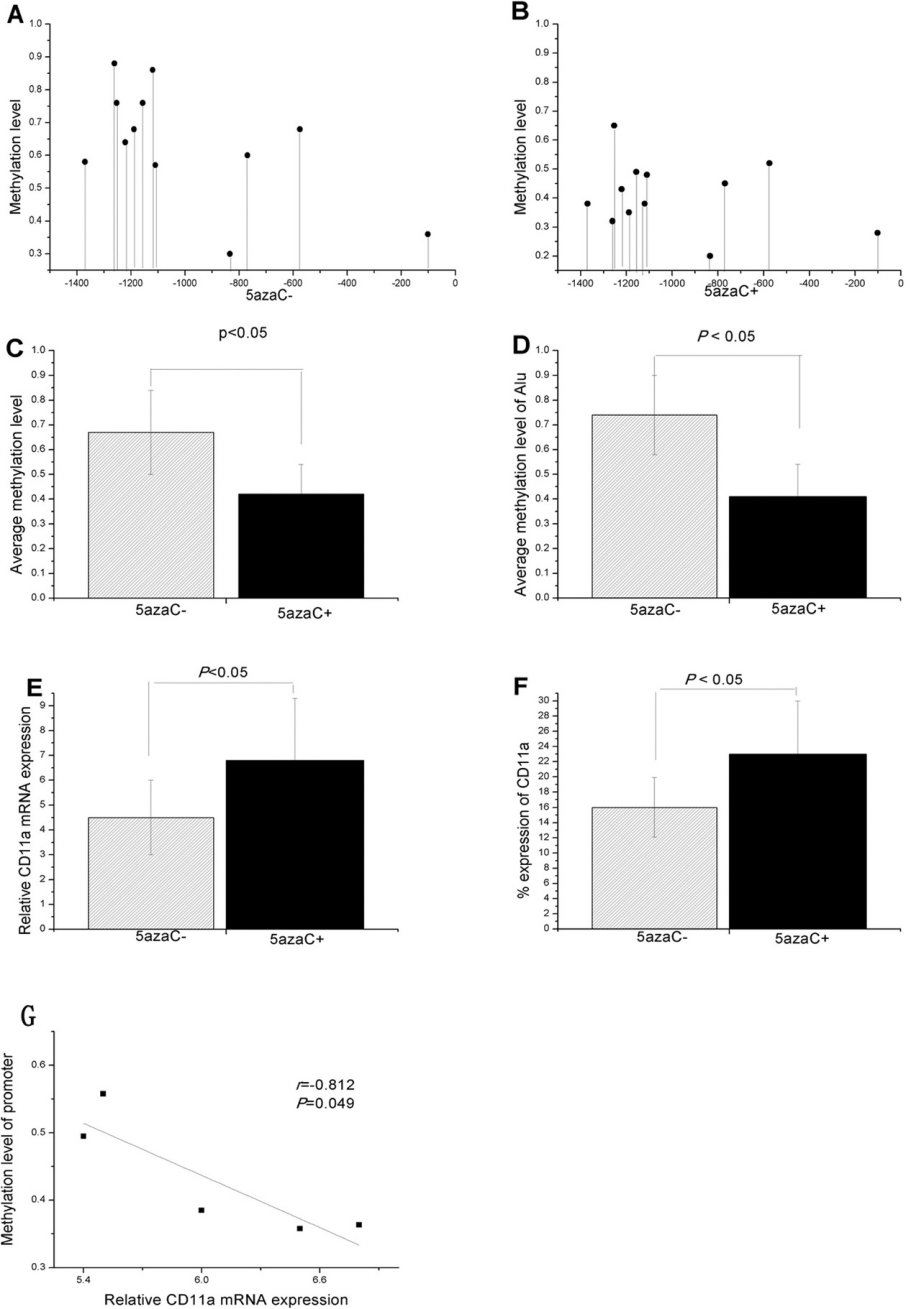
**Treatment with 5**-**azaC decreased CD11a regulatory sequence methylation and increased CD11a expression in normal CD4** ^**+**^ **T cells.** (**A** and **B**) CD11a promoter methylation patterns in **(A)** untreated CD4^+^ T cells and **(B)** 5-azaC-treated CD4^+^ T cells from five healthy subjects. (**C** and **D**) Treatment of CD4^+^ T cells with 5-azaC caused significant **(C)** demethylation of the same promoter region accompanied by **(D)** demethylation of Alu element relative to untreated CD4^+^ T cells. (**E** and **F**) Both **(E)** mRNA and **(F)** protein expression of CD11a were more pronounced in 5-azaC-treated CD4^+^ T cells than in untreated CD4^+^ T cells. **(G)** The mean DNA methylation levels of the CD11a promoter were inversely correlated with the relative mRNA level in 5-azaC treated CD4^+^ T cells.

RT-PCR and flow cytometric analysis was performed to confirm whether DNA methylation directly modifies the ITGAL promoter or regulates CD11a expression by bisulfite sequencing. Normal CD4^+^ T cells were untreated or treated with 5-azacytidine (5-azaC) for 3 days. Figure 3A,B shows the methylation patterns of this region in untreated and 5-azaC-treated CD4^+^ T cells from five healthy subjects. Treatment of CD4^+^ T cells with 5-azaC caused significant hypomethylation of the same promoter region (Figure 3C). This was accompanied by hypomethylation of the Alu element compared to untreated CD4^+^ T cells (Figure 3D).

CD11a expression in these cells was compared. Expression of both mRNA and protein of CD11a was higher within the 5-azaC-treated cells (*P* =0.034 for mRNA and *P* =0.048 for protein, respectively) than in untreated CD4^+^ T cells (Figure 3E,F). CD11a mRNA levels were negatively correlated with the mean methylation status of the promoter region in the 5-azaC-treated cells (Figure 3G).

### CD11a affected the proliferative response of CD4^+^ T cells to autologous PBMCs

**Figure 4:**
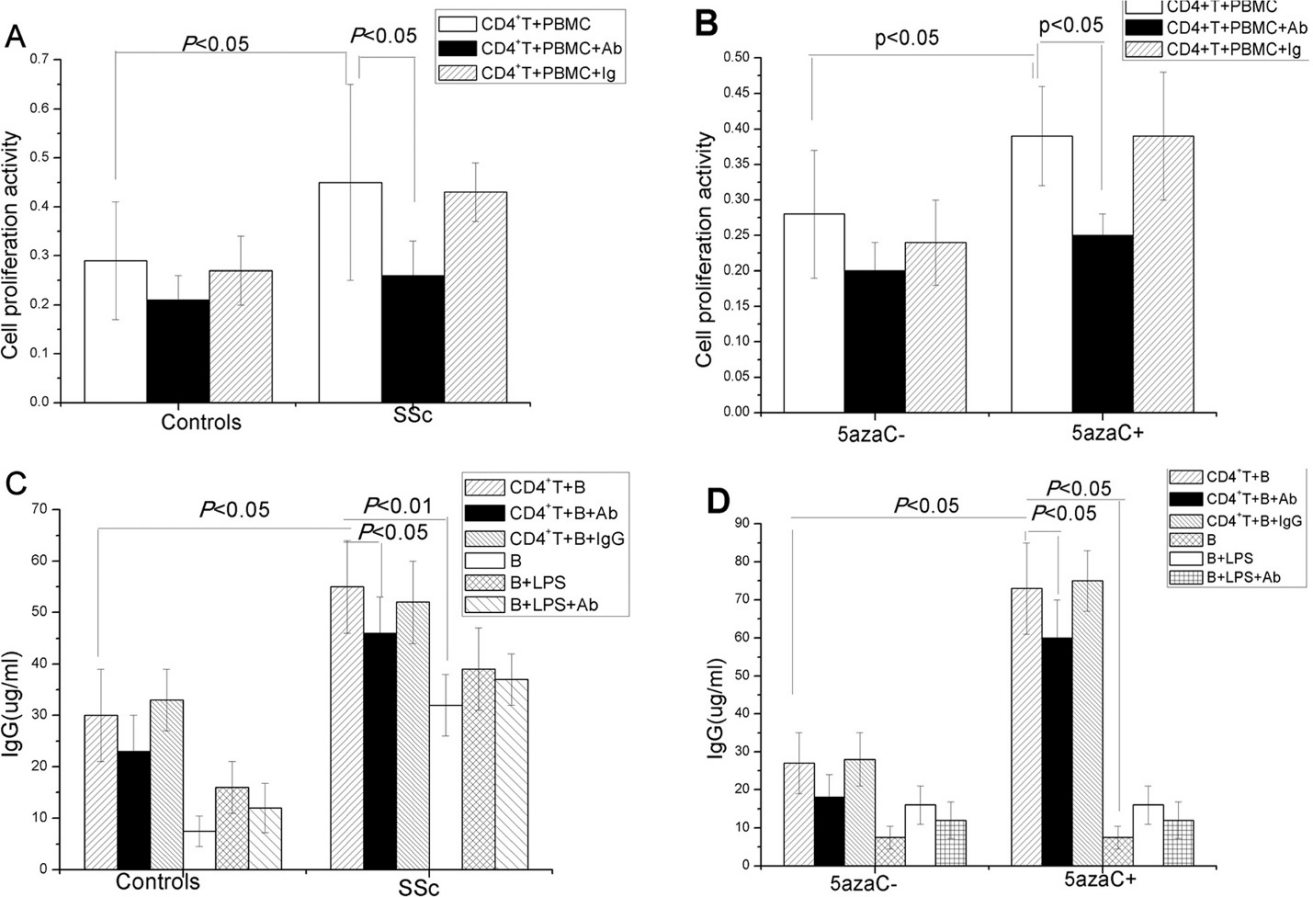
**CD11a overexpression in CD4** ^+^ **T cells increased proliferative response to autologous PBMCs and enhanced production of IgG by co** **-** **cultured B cells. (A)** SSc CD4^+^ T cells significantly increased proliferative response to autologous PBMCs than normal CD4^+^ T cells. **(B)** 5-azaC-treated CD4^+^ T cells showed greater proliferative response to autologous PBMCs than untreated CD4^+^ T cells. (**A** and **B**) The neutralizing antibodies to CD11a markedly decreased proliferative response of SSc CD4^+^ T cells and 5-azaC-treated CD4^+^ T cells but its isotype control, IgG, did not. **(C)** The IgG production was significantly higher in B cells co-cultured with autologous CD4^+^ T cells from SSc patients than in normal CD4^+^ T cells. **(D)** Co-culture of untreated and 5-azaC-treated CD4^+^ T cells with autologous B cells demonstrated enhanced production of IgG by B cells co-cultured with 5-azaC-treated CD4^+^ T cells. (**C** and **D**) The IgG level yielded by B cells cultured alone was significantly lower than that by **(C)** B cells co-cultured with SSc CD4^+^ T cells or **(D)** B cells co-cultured with 5-azaC-treated CD4^+^ T cells. Anti-CD11a mAb, dramatically diminished IgG level produced by autologous B cells co-cultured with SSc CD4^+^ T cells or 5-azaC-treated CD4^+^ T cells but its isotype control IgG did not.

To assess the effects of overexpressing CD11a on the proliferation of SSc CD4^+^ T cells and 5-azaC-treated CD4^+^ T cells, normal CD4^+^ T cells and SSc CD4^+^ T cells were cultured with autologous peripheral blood mononuclear cells (PBMCs). SSc CD4^+^ T cells showed significantly higher proliferative response to autologous PBMCs (as antigen-presenting cells) without antigen than normal CD4^+^ T cells. The neutralizing antibodies to CD11a (anti-CD11a monoclonal antibody (mAb)) markedly decreased the proliferative response of SSc CD4^+^ T cells. The isotype control IgG showed no inhibitory effect (Figure 4A). Similarly, 5-azaC-treated CD4^+^ T cells showed greater proliferation than untreated CD4^+^ T cells. Anti-CD11a mAb, but not the isotype control IgG, dramatically reduced the proliferation of 5-azaC-treated CD4^+^ T cells (Figure 4B).

### CD11a overexpressed on CD4^+^ T cells induced IgG production by autologous B cells

Because CD11a participates in T cell-dependent B cell stimulation, the effect of CD11a on IgG production by B cells was examined. CD4^+^ T cells from SSc patients or healthy subjects were co-cultured with autologous B cells with or without anti-CD11a mAb for 8 days. The amount of IgG in the supernatants was measured by ELISA. As shown in Figure 4C, there was significantly more total IgG production in B cells co-cultured with autologous CD4^+^ T cells from SSc patients than in CD4^+^ T cells from normal subjects. Co-culture of untreated and 5-azaC-treated CD4^+^ T cells and autologous B cells showed more production of IgG by B cells (Figure 4D). Untreated B cells produced significantly less IgG than B cells co-cultured with SSc CD4^+^ T cells or 5-azaC-treated CD4^+^ T cells (*P* <0.05 and *P* <0.05, respectively) (Figure 4C,D). To determine whether CD11a is involved in excessive IgG synthesis by B cells, a blocking anti-CD11a was used in the co-culture of the CD4^+^ T cells with autologous B cells. Anti-CD11a mAb significantly diminished the levels of IgG production by autologous B cells co-cultured with SSc CD4^+^ T cells or 5-azaC-treated CD4^+^ T cells but its isotype control, IgG, did not (*P* <0.05 and *P* <0.05, respectively; Figure 4C,D). A suppressive effect of anti-CD11a mAb on B cells was unlikely because the same amount of anti-CD11a yielded no significant reduction of IgG production by purified B cells stimulated with lipopolysaccharide (*P* >0.05).

### CD11a overexpressed on CD4^+^ T cells induced COL1A2 mRNA expression by normal fibroblasts

**Figure 5:**
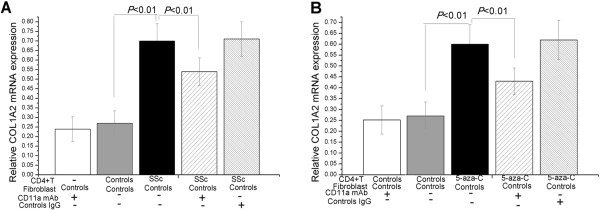
**CD11a overexpressed on CD4** ^+^ **T cells induced COL1A2 mRNA expression by normal fibroblasts. (A)** Significant increases in COL1A2 mRNA expression were observed in normal fibroblasts co-cultured with CD4^+^ T cells from SSc patients (n =5) than in normal controls (n =5). **(B)** There was also a significant increase of COL1A2 mRNA expression in normal fibroblasts co-cultured with 5-azaC-treated CD4^+^ T cells (n =5) relative to untreated CD4^+^ T cells (n =5). (**A** and **B**) The anti-CD11a mAb markedly reduced COL1A2 mRNA transcription in normal fibroblasts stimulated by SSc CD4^+^ T cells and 5-azaC-treated CD4^+^ T cells, but its control, IgG, did not.

The effect of CD11a expressed on SSc CD4^+^ T cells or 5-azaC-treated CD4^+^ T cells on collagen expression in normal fibroblasts was investigated. Different groups of CD4^+^ T cells were co-cultured with normal fibroblasts with or without anti-CD11a antibody for 3 days. Significant increases in COL1A2 mRNA expression were observed in normal fibroblasts co-cultured with CD4^+^ T cells from SSc patients than those from normal controls (*P* <0.01; Figure 5A). There was also a significant increase of COL1A2 mRNA expression in fibroblasts co-cultured with 5-azaC-treated CD4^+^ T cells compared to untreated CD4^+^ T cells (*P* <0.01; Figure 5B). This is the first demonstration that 5-azaC-treated CD4^+^ T cells induce the COL1A2 expression by fibroblasts. The mechanisms underlying these pro-fibrotic actions remain to be identified. The anti-CD11a mAb markedly reduced COL1A2 mRNA transcription in normal fibroblasts stimulated by SSc CD4^+^ T cells and 5-azaC-treated CD4^+^ T cells, but the control IgG did not (*P* <0.01 and *P* <0.01, respectively; Figure 5A,B).

## Discussion

The interaction of LFA-1 (CD11a/CD18) and its ligands provides essential adhesive and co-stimulatory signals required for the initiation of immune responses [8, 9]. The current study demonstrated that CD11a was overexpressed in SSc CD4^+^ T cells, as indicated by RT-PCR analysis and flow cytometric analysis, consistent with previous findings [9, 10]. Expression levels of CD11a were further found to be positively correlated with the extent of clinical disease activity. However, little is known about the mechanisms underlying the overexpression of CD11a in CD4^+^ T cells and its pathogenic role in SSc.

In recent years, accumulating evidence has demonstrated that DNA methylation modifications are reversible, specific to cell type, and strongly involved in the development of autoimmune diseases [24]. DNA hypomethylation of specific regulatory elements leads to overexpression of perforin in CD4^+^ T cells, contributing to monocyte killing in systemic lupus erythematosus [25]. It was here observed that global DNA hypomethylation and levels of the methylation-related modification enzyme DNMT1 were significantly lower in SSc CD4^+^ T cells [26]. It was previously demonstrated that demethylation of CD40L regulatory elements on the inactive X chromosome contributes to CD40L overexpression in CD4^+^ T cells from female SSc patients. This explains, at least in part, the striking predilection of the disease to female patients [27]. It was herein shown that the demethylation of the CD70 promoter region contributes to the overexpression of CD70 in CD4^+^ T cells and may contribute to autoimmune response in SSc [28]. CD11a may also be demethylated in SSc CD4^+^ T cells, suggesting that the methylation status of this gene in CD4^+^ T cells from patients with SSc merited further study.

The methylation level of 23 CG pairs in the promoter region of the CD11a locus in CD4^+^ T cells from patients with SSc was analyzed. Results showed that the average methylation status of CD11a promoter was significantly lower in SSc CD4^+^ T cells and that the degree of methylation was inversely correlated with disease activity. CD11a mRNA levels were negatively correlated with the mean methylation level in the promoter region. Treatment of normal CD4^+^ T cells with 5-azaC decreased the methylation level of CD11a at the same regulatory sequence and increased the level of the CD11a transcriptional activity, indicating the contribution of the same regulatory sequences to CD11a overexpression. The CD11a gene promoter (-1486 to +213) region contains binding sites for several transcription factors including PU1, Sp1, RUNX3, RUNX1/AML-1, C/EBP, ets-related factor, PEBP2/CBF/AML family, and Alu elements which are known to be associated with CD11a transcription, suggesting that the demethylation of the CD11a promoter potentially enhanced its interaction with these transcription factors and the function of Alu elements [29–35]. Collectively, the results of this methylation study demonstrate, for the first time, that DNA demethylation of CD11a promoter sequences contributes to CD11a overexpression in CD4^+^ T cells from patients with SSc.

Although a correlation was observed between demethylation of CD11a promoter regions in association with CD11a overexpression, the molecular mechanisms underlying this demethylation are largely unknown. To date, many studies have shown that inflammation is a key molecular mechanism underlying several chronic autoimmune diseases, affecting DNA methylation on both global and locus-specific levels. This epigenetic modification can be a consequence of inflammatory responses. Future replication studies are warranted to confirm the exact mechanism of demethylation of CD11a promoter regions. There is some concern that the same observations could be reproduced in the affected tissues. Further studies of epigenetic changes in skin lesions and other internal organs would be valuable to determine disease pathogenesis.

Herein, the functional significance of up-regulation of CD11a genes was studied further by demethylation of CD4^+^ T cells in the pathogenesis of SSc. This may facilitate understanding of the etiopathological role of this molecule.

Previous studies have shown that CD4^+^ T cells in SSc present activated phenotypes and are more numerous than those of healthy controls [1, 5, 6]. However, the mechanisms leading to abnormal activation of CD4^+^ T cells in SSc remain largely unknown. Binding of ICAM-1 to LFA-1, the so-called immunological synapse between an activated antigen-presenting cell and a CD4^+^ T cell, is required for full CD4^+^ T cell activation and proliferation [7, 8]. It was here revealed that the proliferative response of SSc CD4^+^ T cells or 5-azaC-treated CD4^+^ T cells to syngeneic PBMCs was significantly increased without exogenous antigen. These processes were significantly suppressed by the anti-CD11a-blocking mAbs, suggesting that the role of CD11a in CD4^+^ T cell activation and proliferation is essential in SSc patients. Previous studies indicated that CD11a overexpression by CD4^+^ T cells affects the development of autoreactivity *in vitro* and autoimmunity *in vivo* [22, 23]. Collectively, these findings and those of related studies confirm that the abnormal activation and proliferation of CD4^+^ T cells attributed to the overexpression of CD11a might be involved in T cell autoimmunity in SSc patients.

The role of the interaction of CD4^+^ T cells with B cells in driving the synthesis and secretion of autoantibodies in SSc is well documented. The presence of a number of different autoantibodies in the serum is a common feature of the disease [36]. LFA-1/ICAMs interaction transduces biochemical signals for T-cell-dependent B cell activation and immunoglobulin production [37–42]. For this reason, whether CD4^+^ T cells could stimulate B cells to produce antibodies through CD11a in SSc was investigated. Results showed that SSc CD4^+^ T cells stimulated IgG production by autologous B cells more robustly than normal CD4^+^ T cells. The increase in IgG synthesis was significantly abrogated by anti-CD11a mAb. In accordance with this, 5-azaC-treated CD4^+^ T cells stimulated autologous B cells to produce IgG. This stimulation was blocked by anti-CD11a antibody. CD11a overexpression provides sufficient B cell stimulatory signals to activate autologous B cells and autoantibody synthesis without any T cell stimulation and antigen presentation in SSc and *in vitro*. In this experiment, anti-CD11a mAb did not completely block IgG production by B cells co-cultured with SSc CD4^+^ T cells or 5-azaC-treated CD4^+^ T cells. It is possible that these CD4^+^ T cells could also stimulate B cells via other signaling pathways. The present results suggest that IgG overproduction by auxiliary B cells may be due, in part, to overexpression of CD11a on CD4^+^ T cells in SSc. Further, there was more total IgG production in purified B cells cultured alone from SSc patients than in purified B cells from normal subjects. The mechanisms leading to higher IgG secretion in SSc B cells are not well understood. It is possible that these B cells play an important role in SSc by a mechanism dependent on antibodies but independent of T cells; the mechanism remains to be elucidated.

Activation of adjacent fibroblasts by CD4^+^ T cells is thought to be critical to the pathological fibrosis in SSc [1, 4, 5]. The mechanism of CD4^+^ T-cell-induced fibrosis merits further examination. Because CD11a plays a role in cell contact between activated CD4^+^ T cells and other cells, including fibroblasts, a question is raised whether CD11a overexpressed on CD4^+^ T cells activates fibroblasts or enhances collagen synthesis. The expression of COL1A2 mRNA in fibroblasts co-cultured with SSc CD4^+^ T cells and normal CD4^+^ T cells was examined. SSc CD4^+^ T cells have a more potent ability to activate fibroblasts than normal CD4^+^ T cells; this increases the rate of COL1A2 mRNA expression. The current results are generally consistent with those of previous studies in which inflammatory cells, especially CD4^+^ T cells, provide important stimuli that drive collagen synthesis in fibroblasts from patients with SSc [4, 5]. CD11a is involved in this process because the anti-CD11a blocking antibody markedly reduced the COL1A2 mRNA expression in fibroblasts co-cultured with SSc CD4^+^ T cells. Fibroblasts were also co-cultured with 5-azaC-treated and control CD4^+^ T cells. Here, 5-azaC-treated CD4^+^ T cells were found to activate fibroblasts to foster COL1A2 mRNA production more strongly than in control CD4^+^ T cells. This production was inhibited by anti-CD11a antibody, as expected. The current work is the first to demonstrate the direct association of CD11a overexpressed on SSc CD4^+^ T cells with excessive expression of COL1A2 by fibroblasts, a considerably important process in the pathogenesis of SSc.

## Conclusions

In conclusion, it is demonstrated herein that CD11a is overexpressed by the demethylation of CD11a promoter regions in SSc CD4^+^ T cells resulting in increased proliferation of CD4^+^ T cells, IgG overproduction by B cells, and excessive collagen synthesis by fibroblasts. Current data provide a new insight into the pathogenesis of SSc and a therapeutic approach via methylation status of CD11a in CD4^+^ T cells.

## Methods

### Subjects

Eighteen SSc patients (10 women and 8 men, mean ± SD age 43 ± 6 years) were recruited from the clinic of the Department of Dermatology, the Second Xiangya Hospital at the Central South University. All patients met the American College of Rheumatology criteria for SSc [43]. Fifteen healthy subjects (8 women and 7 men, mean ± SD age 40 ± 5 years) were recruited from the medical staff at the Second Xiangya Hospital. Patients and healthy subjects were age-, race-, and sex-matched in all experiments. A skin thickening assessment was evaluated using the modified Rodnan total skin score and disease activity was assessed with the SDAI [44, 45]. This study was approved by the Human Ethics Committee of the Central South University Xiangya Medical College. Signed informed consent was obtained from all subjects. The clinical and laboratory characteristics of the patients are shown in Table 1.

**Table 1:** **The clinical and laboratory characteristics of SSc patients**

Patients/Age/Sex	SSc subtype	Disease duration	Clinical features	Current treatment	ANA type	MRTSS	SDAI
1/52/F	lcSSc	2 years	A, B, R	Prednisone, 10 mg/day	Nucleolar	26	3.5
2/45/M	dcSSc	2.5 years	A, E	Prednisone, 12.5 mg/day	Anti-Scl70	33	4
3/43/F	lcSSc	3.5 years	E, M, R	None	ACA	22	2
4/45/M	dcSSc	2.5 years	M, PF, R	Chinese herbal medicines	Anti-RNP	25	3
5/32/F	dcSSc	0.5 years	C, R	None	Anti-Scl70	14	2
6/46/F	dcSSc	1 years	C, E, R	Prednisone, 7.5 mg/day	Nucleolar	39	3.5
7/49/F	lcSSc	2.5 years	A, PF, R	Methylprednisone, 8 mg/day	Nucleolar	28	4.5
8/44/M	dcSSc	2 years	A, E	Methylprednisone, 4 mg/d	Anti-PM-1	18	2
9/47/M	dcSSc	2 years	A, B, PF, R	Chinese herbal medicines	Anti-Scl70	29	5
10/37/F	dcSSc	3 years	A, B, E, PHT, R	Prednisone, 12.5 mg/day	Anti-Scl70	45	9
11/39/M	lcSSc	1.5 years	E, R	None	none	21	2
12/41/F	lcSSc	2 years	A, B, E	Prednisone, 15 mg/day	ACA	27	3.5
13/57/F	dcSSc	2 years	A, E, PF, R	Prednisone, 12.5 mg/day	ACA	40	6
14/43/M	dcSSc	2 years	E, PF, R	None	ACA	36	3
15/35/F	dcSSc	0.5 years	C, E, R	Prednisone, 7.5 mg/day	ACA	22	3
16/36/M	lcSSc	2 years	C, M, R	None	ACA	19	2.5
17/40/F	dcSSc	3 years	A, E, R	Chinese herbal medicines	Anti-RNP	22	4
18/39/M	dcSSc	2.5 years	A, E, M, R	None	Nucleolar	27	6

F, Female; M, Male; dcSSc, Diffuse cutaneous systemic sclerosis; lcSSc, Limited cutaneous systemic sclerosis; A, Arthritis; C, Calcinosis; B, Bowel involvement; E, Esophageal involvement; M, Myositis; PF, Pulmonary fibrosis; PHT, Pulmonary hypertension; R, Reynaud’s phenomenon; ACA, Anticentromere antibodies; MRTSS, Modified Rodnan total skin score; SDAI, Valentini scleroderma disease activity index; ANA type, Anti-nuclear antibodies type; Anti-Scl70, Anti-DNA-topoisomerase I antibodies; Anti-RNP, Anti = nuclear ribonucleic acid protein atibodies; Anti-PM-1, Anti-polymyositis-1 antibodies.

### Cell isolation and culture

#### *CD4*^+^*T cell and B cell isolation*

PBMCs were isolated by Ficoll-Hypaque density gradient centrifugation (Hengxin Chemical Reagent Co, Ltd., Shanghai, China) and CD4^+^ T cells and B cells were isolated by positive selection using magnetic beads according to the manufacturer’s protocol (Miltenyi Biotec, Bergisch Gladbach, Germany). The purity of the enriched immune cell subsets was confirmed to be over 95% by flow cytometry. CD4^+^ T cells and B cells were cultured as previously described [46].

#### *Treatment of CD4*^+^*T cell with a DNA methyltransferase inhibitor*

Normal CD4^+^ T cells were stimulated with 1 μM phytohemagglutinin, treated with 1 μM 5-azaC (DNA methyltransferase inhibitor, Sigma, St. Louis, MO, USA) and cultured for 3 days [46].

#### Fibroblast isolation and culture

Normal dermal fibroblasts were established as described previously [47]. Human fibroblasts were seeded in 12-well plates at a density of 5 × 10^4^ cells/well in Dulbecco’s modified Eagle medium/10% heat inactivated FBS for 48 h to 80% confluence. The fibroblasts between passages 3 and 6 in monolayer culture were used for the experiment. Fibroblasts were evaluated using immunocytochemical staining for cytokeratin and vimentin.

### Flow cytometric analysis

PBMC suspensions (1 × 10^5^ cells) were briefly stained with 20 μL PE-conjugated anti-human CD4 and FITC-conjugated anti-human CD11a antibodies (Becton Dickinson, Franklin Lakes, NJ, USA) for 30 min, and the cells were sorted with a FACScalibur system (Becton Dickinson). The relative number of CD4- and CD11a-positive cells was calculated using CellQuest Software (Becton Dickinson).

### RNA isolation, cDNA synthesis, and real-time RT-PCR

Total RNA was isolated from CD4^+^ T cells and co-cultured fibroblasts using an RNeasy mini kit (Qiagen, Valencia, CA, USA) according to the manufacturer’s instructions and stored at -80°C. First-strand cDNA was synthesized using a RevertAid™ First Strand cDNA Synthesis Kit (Takara, Otsu, Japan). Real-time quantitative RT-PCR was performed using a RotorGene™ 3000 (Corbett Research, Mortlake, NSW, Australia) to quantify the amount of RNA. β-actin was used as an endogenous control to normalize the amount of total RNA. Primers used were as follows. β-actin: forward 5′-GCACCACACCTTCTACAATGAGC-3′ and reverse 5′-GGATAGCACAGCCTGGATAGCAAC-3′; CD11a: forward 5′-TGAGAGCAGGCTATTTGGGTTAC-3′ and reverse 5′-CGGCCCATGTGCTGGTAT-3′; Proalpha 2(1) collagen (COL1A2): forward 5′-GATGTTGAACTTGTTGCTGAGG-3′ and reverse 5′-TCTTTCCCCATTCATTTGTCTT-3′.

### Genomic DNA extraction and bisulfite sequencing

Genomic DNA from the CD4^+^ T cell subset was isolated using a TIANamp Genomic DNA Kit (Tiangen Biotech, Beijing, China). Bisulfite conversion of genomic DNA was performed using an EpiTect Bisulfite Kit (Qiagen, Valencia, CA, USA). The methylation status was analyzed in a 1.7 kb fragment of the CD11a gene promoter sequence containing 23 CG pairs. A fragment of a CD11a promoter sequence including a 589 bp fragment located immediately 5′ to the CD11a gene transcription start site (-376 to +213), a 474 bp fragment (-1015 to -541), and a 492 bp fragment (-1486 to -995) was also used. Fragments were amplified using PCR and cloned into the pGEM-T easy vector (Promega, Madison, WI, USA). Ten independent clones were sequenced for each of the amplified fragments. The following primers were used: fragment (-376 to +213): forward 5′-AAGGTCCAGAGAAAGCTCTCAC-3′ and reverse 5′-CTACACCAAACCCTACAATTTCTC-3′; fragment (-1015 to -541): forward 5′-AAAAAATTGGGTATAGTGGTTT-3′ and reverse 5′-TCTCTTAAAACCAAAAATCAAA-3′; fragment (-1486 to -995): forward 5′-TGTTATTGGAGAAATGTTTATTTAAA-3′ and reverse 5′-AACCACTATACCCAATTTTTTAAA-3′.

### Co-culture of CD4^+^ T cells with autologous PBMCs

CD4^+^ T cells (2 × 10^4^, 100 μL) and irradiated autologous PBMCs (2 × 10^4^, 100 μL), which are antigen-presenting cells, were co-cultured in 96-well plates for four days [46]. Then 1 μg/mL of anti-CD11a mAb or mouse isotype control IgG1 (eBioscience, SanDiego, CA, USA) was used to block the interaction of LFA-1/ICAMs.

### Cell proliferation assays

After 4 days of co-culture of CD4^+^ T cells and autologous PBMCs, cell proliferation was performed by bromodeoxyuridine incorporation using a colorimetric ELISA kit according to the manufacturer’s instructions (Roche Diagnostics, Mannheim, Germany). Absorbance was measured using a microplate reader (Bio-Tek Elx800) at 370 nm with the reference absorbance at 492 nm. Results are given as the mean ± SEM of triplicate determinations.

### CD4^+^ T cells and autologous B cell co-stimulation assay

CD4^+^ T cells (1 × 10^5^, 100 μL) were co-cultured with autologous B cells (4 × 10^5^, 100 μL) in U-bottom 96 well plates (5 × 10^5^, 200 μL volume/well) as described previously [46]. Anti-CD11a mAb, 1 μg/mL, or mouse isotype control IgG1 was used to observe the LFA-1/ICAM blocking role when CD4^+^ T cells and B cells were incubating. B cells alone, B cells plus lipopolysaccharide (Sigma, St Louis, MO, USA), and B cells plus lipopolysaccharide and anti-CD11a were cultured as controls. After incubation in RPMI 1640/10% FBS/penicillin/streptomycin at 37°C/5% CO_2_ for 8 days, 50 μL fresh media was added to each well. On the fourth day after that, supernatants (200 μL) were harvested and stored at 4°C.

### ELISA for IgG

IgG levels in the supernatants of the CD4^+^ T and B cell cultures were detected using an ELISA kit (Columbia Bio) in accordance with the manufacturer’s protocol. All determinations were performed in triplicate.

### Co-culture of CD4^+^ T cells with normal fibroblasts

CD4^+^ T cells and plated fibroblasts were washed three times with PBS. Subsequently, CD4^+^ T cells (1 × 10^4^/well) were added to fibroblast monolayers and culture plates were incubated at 37°C/5% CO_2_ for 3 days as described previously [48]. In some experiments, 1 μg/mL of neutralizing mAb against to CD11a or mouse isotype control IgG1 was added to the co-cultures. After 3 days of co-culture, non-adherent cells were washed out, and only fibroblasts were harvested and analyzed for the COL1A2 mRNA expression. All cultures were established in triplicate.

### Statistical analysis

Data are expressed as the mean ± standard deviation (SD) and all analyses were performed using SPSS 18.0 software (SPSS Inc., Chicago, IL, USA). Group variables were compared using the student *t*-test for continuous variables and Mann-Whitney *U*-test for non-normally distributed data. Correlations were assessed using Pearson’s rank order and Spearman coefficients.

## Abbreviations

5-azaC: 5-azacytidine
LFA-1: Lymphocyte function-associated antigen-1
mAb: Monoclonal antibody
PBMCs: Peripheral blood mononuclear cells
SDAI: Valentini scleroderma disease activity index
MRTSS: Modified Rodnan total skin score
SSc: Systemic sclerosis.
